# Prognostic markers compared to CD3+TIL in locally advanced nasopharyngeal carcinoma

**DOI:** 10.1097/MD.0000000000027956

**Published:** 2021-11-19

**Authors:** Nasser Al-Rajhi, Shamayel F. Mohammed, Hatim A. Khoja, Mohammad Al-Dehaim, Hazem Ghebeh

**Affiliations:** a Department of Radiation Oncology, King Faisal Specialist Hospital and Research Center, Riyadh, Saudi Arabia; b Deparment of Laboratory Medicine and Pathology, King Faisal Specialist Hospital and Research Centre, Riyadh, Saudi Arabia; c Stem Cell & Tissue Re-engineering Program, Research Centre, King Faisal Specialist Hospital and Research Centre, Riyadh, Saudi Arabia.

**Keywords:** albumin-to-alkaline phosphatase, chemo-radiotherapy, EBV copy number in peripheral blood, hemoglobin, nasopharyngeal carcinoma, neutrophils -to-lymphocytes ratio, platelets-to-lymphocytes ratio, tumor-infiltrating CD3+ Lymphocytes

## Abstract

Locally advanced nasopharyngeal carcinoma (LA-NPC) is more prevalent in some geographic regions, including Saudi Arabia. Typically, Tumor-Node-Metastasis (TNM) staging is used in NPC. However, it is inadequate to assess the prognosis of LA-NPC.

Therefore, we analyzed and compared several previously reported prognostic factors in LA-NPC patients, retrospectively, including CD3+tumor-infiltrating lymphocytes (TIL) and peripheral blood hemoglobin, EBV DNA copy number, ratios of albumin-to-alkaline phosphatase ratio (AAPR), neutrophils, or platelets-to-lymphocytes (NLR, PLR). The studied cohort was 83 LA-NPC patients previously recruited for a randomized phase II trial with a different aim.

Univariate cox regression analysis showed no significant correlation between any of the tested variables with disease-free survival (DFS) or overall survival (OS) with the exception of low CD3+ TIL infiltration, which correlated significantly with DFS (HR = 6.7, *P* = <.001) and OS (HR = 9.1, *P* = .043). Similarly, in a validated multivariate cox regression analysis, only low CD3+ TIL correlated significantly with DFS (HR = 7.0, *P* < .001 for TIL) and OS (HR = 9.4, *P* = .040).

Among tested parameters, CD3+ TIL was the only independent prognostic marker for DFS and OS in LA-NPC patients treated with CCRT. This study supports the use of CD3+TIL, over other factors, as an independent prognostic factor in LA-NPC.

## 1 Introduction

Nasopharyngeal Carcinoma (NPC) is sensitive to radiation therapy and chemotherapy. It is a common head and neck malignancy in Southeast Asia, the Middle East and parts of North Africa. Currently, most patients have Locally advanced NPC (LA-NPC) at the time of diagnosis, defined as stage III or IV_._ Despite the advances in therapy, some patients relapse and die of their disease. Biomarkers are needed to predict the response to therapy. Tumor-Node-Metastasis (TNM) staging is currently used to assess the prognosis of NPC. However, it is not adequate to assess the prognosis of LA-NPC.

We have previously found CD3+ TIL as a robust and independent prognostic factor for LA-NPC.^[1]^ Several studies tested other clinicopathological factors as prognostic factors or LA-NPC, including neutrophil-to-lymphocyte ratio (NLR),^[2,3]^ Platelet-to-lymphocyte ratio (PLR),^[4]^ albumin-to-alkaline phosphatase ratio (AAPR),^[5]^ baseline hemoglobin (HG)^[6]^ and EBV DNA copy number in the peripheral blood.^[7]^ The existence of multiple prognostic factors highlights the importance of testing them side-by-side to find the most appropriate markers and their relationship in a specific geographical area.

We recently reported on the nonsignificant effect of low-dose fractionated radiotherapy (LDFRT) on induction chemotherapy followed by concurrent chemo-radiation in LA-NPC.^[8]^ We used this cohort of LA-NPC patients to study the correlation of different prognostic factors with survival.

In the current study, we compared side-by-side different prognostic markers previously reported to correlate with survival in NPC. In our studied cohort, low CD3+TIL was the only independent prognostic factor for LA-NPC.

## 2 Methods

This study is a retrospective, single-institution, cohort study testing the effect of different makers on the survival of NPC patients. The study was a secondary analysis of a randomized controlled trial made for a different aim.^[8]^

This study was conducted according to the declaration of Helsinki and guidelines of the Institutional Review Boards (IRBs) of King Faisal Specialist Hospital and Research Centre (Research Ethics Committee, REC, and Basic Research Committee BRC) who approved the work under RAC# 2150-013. The need for consent has been waived as the study was retrospective and patients were not individually identifiable.

### 2.1 Patient selection

All enrollments were among NPC patients recruited to test the effect of low-dose fractionated radiation with induction neoadjuvant chemotherapy followed by concurrent chemoradiotherapy (CCRT) trial^[8]^ (registered in clinicaltrials.gov: NCT03890185). Among 108 patients enrolled, tissue blocks were only available for 83 patients (42 in the control arm and 41 patients in the experimental arm). According to the 2018 American Joint Committee on Cancer (AJCC 8th edition) staging system, all patients were manually restaged and designated as stage III or IV. The treatment arm “Trial Arm” was part of the multivariate analysis. Patients were followed up regularly in the clinics or by phone.

### 2.2 Neutrophils -to-lymphocytes ratio, platelets-to-lymphocytes ratio, hemoglobin EBV DNA in peripheral blood

Data on NLR, PLR, HG within 2 weeks of diagnosis and before initiation of treatment was available in patients’ medical files. Similarly, EBV DNA status in peripheral blood was available as routine care for NPC patients. Data were missing on EBV DNA status for eight patients. The used cutoff was based on what was used in previous reports (NLR, HG, EBV DNA) or the median (AAPR and PLR).

### 2.3 Immunohistochemistry

Formalin-fixed paraffin-embedded (FFPE) NPC tissue blocks, obtained at the time of diagnosis, were used for this study. Immunostaining was performed as previously described in detail.^[1]^ Briefly, immunohistochemistry was performed on 4 μm sections using a fully automated Ventana Benchmark Ultra (Ventana/Roche) system. The CD3 antibody was a ready-to-use primary antibody from Ventana. Antibody binding was detected using the ultraView DAB detection kit available from Ventana.

### 2.4 Pathological scoring

As adapted from Salgado et al,^[9]^ scoring of lymphocyte infiltration was done in a semi-quantitative estimation giving a 4-tier scale score as previously described in detail.^[1]^ The score depended on the percentage of CD3+ TIL occupying the field. Score 1 was given for TIL occupying <10% of the field, while scores 2, 3, and 4 were given for TIL occupying 10 to 40, 40 to 70, and >70% of the field, respectively. Two independent anatomical pathologists (HK & SM), blinded to the treatment outcome, scored and interpreted the histological tumor sections. A consensus was reached in case of discrepancy. Scores were further dichotomized into low CD+3 TIL (scores 1 and 2) or high CD3+ TIL (scores 3 and 4).

### 2.5 Statistical analysis

The correlation between proposed risk factors and survival outcomes was evaluated using Cox proportional hazard model. The model was further validated by forward selection using the Bayesian information criterion (BIC) validation method. Survival plots were generated using Kaplan–Meier methods, while survival curves were compared using the log-rank test. Time was censored for patients who were alive or disease-free at the last follow-up. All analysis was performed using JMP statistical software (Cary NC, USA), and a *P* value of .05 was the threshold for significance.

## 3 Results

### 3.1 Patient characteristics

The majority of patients were males with large tumors (T3 and T4), advanced lymph node involvement (N), which is consistent with LA-NPC. Tumors were predominantly non-keratinizing undifferentiated carcinoma, that is WHO type III histological subtype (Table 1).

**Table 1:** Patients characteristics.

Features	Categories	# Patients
Age Range 18–72 Median 45	<40 yr ≥ 40 yr	32 ^∗^(39) 51 (61)
Gender	Female Male	21 (25) 62 (75)
WHO Type	I II III	0 (0) 5 (6) 78 (94)
T stage	T1 T2 T3 T4	26 (31) 2 (2) 30 (36) 25 (30)
N stage	N0 N1 N2 N3	5 (6) 12 (15) 24 (29) 42 (51)
TNM staging	III IV	24 (29) 59 (71)
Relapse	No Yes	63 (76) 20 (24)
Type of relapse	None Local Locoregional Systemic	63 (77) 3 (4) 3 (2) 14 (16)
Survival	Alive Dead	76 (92) 7 (8)

∗ Percentage of cases.

LA-NPC patients were followed up for a median time of 4.7 years from the time of diagnosis, of which 20 (24%) relapsed, including 5 patients with local or loco-regional and another 16 (25%) with systemic relapse. From all the LA patients, eventually, 7 (8%) died of the disease.

### 3.2 Correlation between the different clinicopathological markers

Age correlated with CD3+TIL as older patients had lower TIL scores (*P* = .011). Patients with anemia were mostly young (*P* = .047) and females (*P* < .001). There was a correlation between EBV copies >1500 copies/mL and clinical-stage 4A (*P* = .015). On the other hand, AAPR, NLR, and PLR did not correlate significantly with any other tested factor (data not shown).

### 3.3 Correlation of clinicopathological and other biomarkers with survival

We then looked at the correlation of different clinicopathological markers, including CD3+ TIL, AAPR, NLR, PLR, HG, and EBV DNA copies, with the disease outcome. Univariate Cox regression analysis showed that among tested makers, only low CD3+TIL correlated significantly with worse DFS (HR = 6.7, *P* < .001) while there was a trend of correlation with only borderline significance between worse DFS and WHO type II as well as worse DFS and lower AAPR (Table 2). On the other hand, low CD3+ TIL was the only makers that significantly correlated with worse OS (HR = 9.1, *P*.043), while there was a trend of correlation between worse OS and higher NLR (>3) or PLR (>172) with only a borderline significance.

**Table 2:** Univariate Cox proportional hazard regression analysis of the different clinicopathological features and biomarkers with disease-free survival and overall survival in 83 patients with LA-NPC.

	Relapse		DFS			Death		OS		
	−	+	HR	95% CI	^∗^ *P*	−	+	HR	95% CI	^∗^ *P*
Age										
<40 yr	27 (84)^†^	5 (16)	1			30 (94)	2 (6)	1		
≥40 yr	36 (71)	15 (29)	1.98	0.7–5.5	.183	46 (90)	5 (10)	1.5	0.3–8.0	.601
Gender										
Females	18 (86)	3 (14)	1			20 (95)	1 (5)	1		
Males	45 (73)	17 (27)	2.2	0.7–7.7	.197	56 (90)	6 (10)	2.1	0.3–17.8	.472
WHO Type										
III	61 (78)	17 (22)	1			71 (91)	7 (9)	1		
II	2 (40)	3 (60)	3.2	0.9–11.0	.068	5 (100)	0 (0)	0.0001	2.6x10^-105–7.2x10^96	.940
Trial Arm^§^										
Control arm	34 (81)	8 (19)	1			39 (93)	3 (7)	1		
LDXRT	29 (71)	12 (29)	1.7	0.7–4.1	.253	37 (90)	4 (10)	1.5	0.3–6.7	.592
UICC Stage										
III	17 (71)	7 (29)	1			20 (83)	4 (17)	1		
IV	46 (78)	13 (22)	1.4	0.6–3.5	.478	56 (95)	3 (5)	0.3	0.1–1.2	.090
CD3+TIL										
High	**43 (91)**	**4 (9)**	**1**			**46 (98)**	**1 (2)**	**1**		
Low	**20 (56)**	**16 (44)**	**6.7**	**2.2–20.2**	**< .001**	**30 (83)**	**6 (17)**	**9.1**	**1.1–76.1**	**.042**
AAPR										
>0.58	34 (85)	6 (15)	1			36 (90)	4 (10)	1		
<0.58	29 (67)	14 (33)	2.4	0.9–6.3	.076	40 (93)	3 (7)	0.7	0.2–3.2	.671
NLR										
< 3	47 (78)	13 (22)	1			57 (95)	3 (5)	1		
> 3	16 (70)	7 (30)	1.6	0.6–3.9	.341	19 (83)	4 (17)	7.6	0.8–17.0	.083
PLR										
<172	36 (80)	9 (20)	1			44 (98)	1 (2)	1		
> 172	27 (71)	11 (29)	1.5	0.6–3.7	.334	32 (84)	6 (16)	1.8	0.8–86.1	.074
HG										
Low (<110 g/L)	20 (87)	3 (13)	1			22 (96)	1 (4)	1		
Normal (≥110 g/L)	43 (72)	17 (28)	2.4	0.7–8.2	.160	54 (90)	6 (10)	2.4	0.3–19.8	.421
EBV DNA copies^‡^										
Negative (< 1500 copies/mL)	46 (75)	15 (25)	1			55 (90)	6 (10)	1		
Positive (≥1500 copies/mL)	10 (71)	4 (29)	1.2	0.4–3.5	.773	13 (93)	1 (7)	0.7	0.1–6.0	.761

(+ and -) are numbers patients.

∗ *P* values in bold represent significant data.

† Numbers between brackets are the percentages of patients.

‡ Eight samples had unknown EBV DNA copies status.

§ Trial arm in a previous trial (NCT03890185) that is not related to this study where Control arm = No irradiation during neoadjuvant chemotherapy while LDXRT = Lose dose of irradiation during the neoadjuvant chemotherapy. AAPR = albumin-to-alkaline phosphatase ratio, NLR = neutrophils-to-lymphocyte ratio, PLR = platelets-to-lymphocytes ratio.

**Figure 1:**
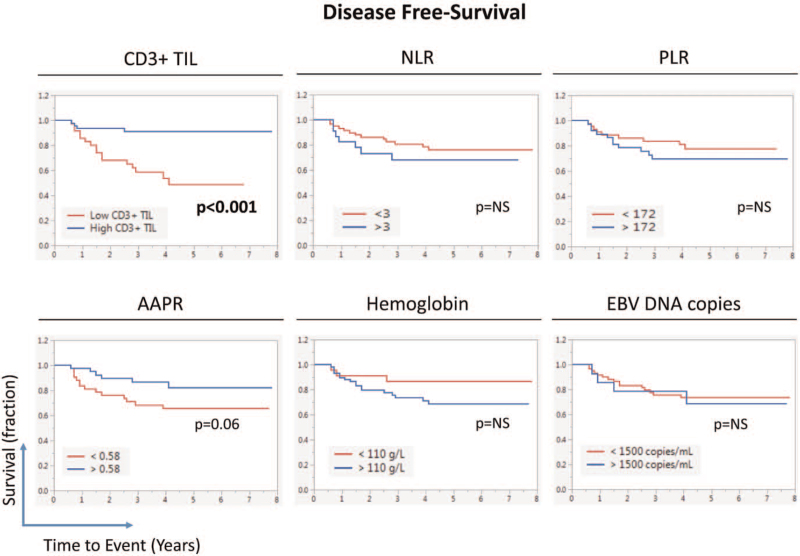
Effect of various Biomarkers on the disease-free survival (DFS) of LA-NPC patients (n = 83). Kaplan–Meier survival curves showing disease-free survival (DFS) of patients in relation to their Tumor CD3+TIL infiltration or peripheral blood's NLR (Neutrophil-to-Lymphocytes Ratio), PLR (Platelet-to-Lymphocytes Ratio), (Albumin-to- Alkaline Phosphatase Ratio) AAPR, hemoglobin, or EBV DNA copies. Statistical significance was calculated using the log-rank test.

**Figure 2:**
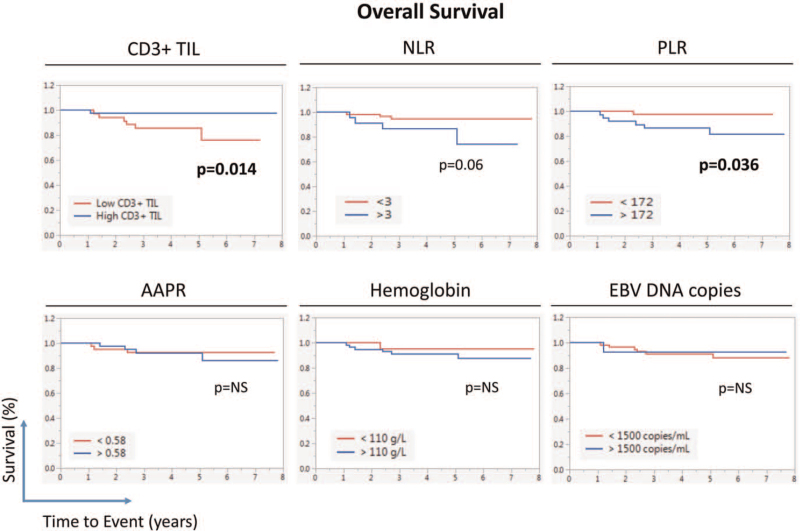
Effect of various Biomarkers on the overall survival (OS) of LA-NPC patients (n = 83). Kaplan–Meier survival curves showing disease-free survival (DFS) of patients in relation to their Tumor CD3+TIL infiltration or peripheral blood's NLR (Neutrophil-to-Lymphocytes Ratio), PLR (Platelet-to-Lymphocytes Ratio), (Albumin-to- Alkaline Phosphatase Ratio) AAPR, hemoglobin, or EBV DNA copies. Statistical significance was calculated using the log-rank test.

Kaplan–Meier survival curves showed statistically significant separation between DFS of low and high CD3+ TIL groups of patients (Fig. 1, log-rank *P* < .001). On the other hand, the significance of the difference in DFS between low and high AAPR was borderline. Similarly, Kaplan-Meier survival curves showed statistically significant separation between OS of low and high CD3+ TIL groups of patients (log-rank *P* .015, Fig. 2). Similarly, there was a significant separation between OS of patients with high and low PLR (log-rank, *P* .036).

Multivariate Cox regression analysis was performed to determine independent factors that correlate with the survival of LA-NPC in our patients. While both CD3+TIL and AAPR correlated significantly with DFS (*P* = .004 and *P* = .017 respectively), the significance of the correlation remained only with CD3+TIL and DFS upon validation (HR = 5.9, *P* < .001) (Table 3). On the other hand, there was no significant correlation between any of the tested factors and OS. However, upon validation, low CD3+TIL significantly correlated with shorter DFS (HR = 7.0, *P* < .001) and OS (HR = 9.4, *P* < .001), and there was borderline significance between higher PLR and shorter OS (Table 4). Altogether, CD3+ TIL was the only independent prognostic factor that correlated significantly with DFS and OS in validated multivariate analysis.

**Table 3:** Multivariate Cox proportional hazard survival analysis for clinicopathological features and other biomarkers as disease-free survival and overall survival in 83 patients with LA-NPC.

	DFS			OS		
	HR	95% CI	^∗^ *P*	HR	95% CI	*P*
Age			.422			.736
Gender			.638			.738
WHO Type			.201			.922
Trial Arm			.509			.927
UICC Stage			.207			.236
CD3+TIL	6.0	1.7–21.0	**.005**	8.2	0.7–97.0	.095
AAPR	3.2	1.1–10.0	**.040**			.651
NLR			.908			.446
PLR			.122			.184
HG			.546			.917
EBV DNA copies			.400			.444

∗ ***P*** values in bold represent significant data of the multivariate analysis before validation.

**Table 4:** Validated Multivariate Cox proportional hazard survival analysis for clinicopathological features and other biomarkers as disease-free survival and overall survival in 83 patients with LA-NPC.

	DFS			OS		
	HR	95% CI	*ν P*	HR	95% CI	*ν P*
Age			1.000			1.000
Gender			1.000			1.000
WHO Type			1.000			1.000
Trial Arm			1.000			1.000
UICC Stage			1.000			1.000
CD3+TIL	6.9	2.3–21.1	**<0.001**	9.6	1.1–81.3	**0.038**
AAPR			1.000			1.000
NLR			1.000			1.000
PLR			1.000	6.8	0.81–56.1	0.077
HG			1.000			1.000
EBV DNA copies			1.000			1.000

***νP*** represents the significant data after validation using forward selection and the Bayesian information criterion (BIC) validation method.

## 4 Discussion

Despite the importance of TNM staging in cancer, it is less helpful in LA-NPC, an advanced form of the disease that is common in some geographical regions like Saudi Arabia. We have previously shown that among several immunological markers, CD3+ TIL was the most promising as a robust and practical marker to estimate the prognosis of LA-NPC in our patients. In this work, we have compared CD3+TIL with other clinicopathological factors commonly associated with LA-NPC or NPC's survival in general. Although there was a trend for correlation between AAPR, NLR or PLR and survival, CD3+TIL was the only significant and independent prognostic factor for LA-NPC in multivariate analysis.

Albumin is the most abundant protein in the blood, and its level correlates with the nutritional status of patients.^[10]^ In addition, several tumor-released cytokines like TNF-α and IL-6 can module albumin levels; thus, it reflects indirectly on the patient's tumor status. On the other hand, alkaline phosphatase is commonly released into the bloodstream upon bone metastasis.^[11]^ Therefore, AAPR has been recently used as a prognostic marker in several malignancies, including NPC.^[5,12]^ In our cohort of LA-NPC, there was a trend for a correlation between AAPR and RFS; however, it did not reach statistical significance.

Neutrophils and lymphocytes constitute a large portion of circulating white blood cells. The general state of a cancer patient can affect the expansion, activation status, or distribution of peripheral blood cells’ various components, especially neutrophils and lymphocytes. Stress hormones like glucocorticoids and catecholamine have an important complex impact on the distribution of leukocytes and their subtypes between blood, bone marrow, and tissues (Reviewed by Ince et al ^[13]^). Glucocorticoids can induce apoptosis of lymphocytes,^[14]^ while adrenaline can increase lymphocyte number.^[13]^ Although the relationship is complex, higher neutrophils in tumors in many cases can reflect a failed immune response as they promote tumor growth and metastasis, while they can have an antitumor effect in other situations/cancers.^[15]^ NLR is associated with a worse prognosis in many cancers, including breast,^[16]^ esophageal,^[17]^ ovarian,^[18]^ and lung^[19]^ cancers, as well as Hodgkin's lymphoma.^[20]^ Similarly, higher NLR has been associated with a poorer prognosis in NPC.^[2,3]^ However, many of these reports were meta-analyses that pooled data from several studies to increase sample size and show the trend and its significance. In the current study, we also found a trend of lower OS of LA-NPC patients with higher NLR while the significance was borderline (Fig. 2). It is possible that in larger cohorts, the statistical significance would be reached.

Platelets in the blood can facilitate tumor cell metastasis through multiple mechanisms (Reviewed by Schlesinger et al ^[21]^). Platelets can encase tumor cells in the bloodstream and protect them from natural killer cells. Furthermore, platelets can attract granulocytes to facilitate tumor cells’ extravasation from the bloodstream to form a metastatic colony. The platelet-to-lymphocyte ratio (PLR) is an inflammatory marker applied for many diseases, including cancer. PLR is associated with a worse prognosis of many cancers.^[22]^ Similarly, meta-analyses from a pool of several studies have shown that PLR is associated with poor prognosis in NPC.^[4,23]^ In the current study, higher PLR was associated with shorter OS (*P* = .036, Fig. 2); however, the association was not statistically significant in multivariate analysis.

Generally, lower baseline hemoglobin (<110 g/L) is associated with a worse status of patients in NPC;^[6]^ however, there was no significant correlation of baseline hemoglobin with the survival of NPC patients in this study. As expected, lower baseline hemoglobin was associated with young and female patients, factors that tend to be associated with better survival making the association between hemoglobin and NPC complicated in countries with relatively young patients like Saudi Arabia.

The EBV DNA copy number in blood post-treatment is associated with a worse prognosis in NPC;^[7]^ however, there was no correlation between EBV DNA copy and survival in our study. One of our study's limitations was the missing EBV DNA copy status in 8 samples, likely affecting the correlation results. This is in addition to other limitations including, being a secondary analysis for a trial that was done for a different aim, limitation in factors that could be analyzed and an experimental treatment arm not related to this study. However, the last issue was considered in the multivariate analysis.

The fact that CD3+TIL correlated significantly with DFS and OS in univariate and multivariate analysis in this cohort of a relatively small number of patients likely suggests that CD3+ TIL as a biomarker has higher prognostic power than other factors. This does not exclude that the other markers would be prognostic in larger cohorts of patients.

Altogether, there was a trend of correlation between higher AAPR and worse DFS and higher NLR and PLR with worse OS. However, in multivariate analysis, the CD3+ TIL correlation with DFS and OS was the only statistically significant correlation supporting CD3+ TIL as an independent prognostic factor for LA-NPC.

## 5 Conclusion

Among several makers tested, CD3+TIL was the only significant and independent prognostic marker for LA-NPC in this study.

## Acknowledgments

We would like to thank King Abdulaziz City for Science and Technology (KACST) for supporting this work financially under the national science and technology NSTP project #12-MED2423-20 (Nasser Al-Rajhi).

## Author contributions

**Conceptualization:** Nasser Al-Rajhi, Hazem Ghebeh.

**Data curation:** Nasser Al-Rajhi, Shamayel F. Mohammed, Hatim A. Khoja, Mohammad Al-Dehaim, Hazem Ghebeh.

**Formal analysis:** Hazem Ghebeh.

**Funding acquisition:** Nasser Al-Rajhi.

**Investigation:** Hazem Ghebeh.

**Methodology:** Hazem Ghebeh.

**Project administration:** Hazem Ghebeh.

**Software:** Hazem Ghebeh.

**Supervision:** Hazem Ghebeh.

**Writing – original draft:** Hazem Ghebeh.

**Writing – review & editing:** Nasser Al-Rajhi, Hazem Ghebeh.
